# Cylindrical vector beams demultiplexing communication based on a vectorial diffractive optical element

**DOI:** 10.1515/nanoph-2023-0009

**Published:** 2023-03-17

**Authors:** Mengwei Cao, Zhenwei Xie, Yanan Zhong, Ting Lei, Wanlong Zhang, Shutian Liu, Xiaocong Yuan

**Affiliations:** Department of Physics, Harbin Institute of Technology, Harbin, 150001, China; Nanophotonics Research Center, Institute of Microscale Optoelectronics & State Key Laboratory of Radio Frequency Heterogeneous Integration, Shenzhen University, Shenzhen, 518060, China

**Keywords:** cylindrical vector beams, Demultiplexing, metasurface, PB-phase

## Abstract

Cylindrical vector beams with polarization singularities, transmission stability and turbulence resilient, are orthogonally structured light beams providing new degrees of freedom for multiplexing optical communications. The demultiplexing of the CVBs with high efficiency and low crosstalk is of vital importance for the practical applications. Here, we propose a lens-less CVB sorting approach with a set of dielectric metasurface devices. The metasurface is composed of elliptical silicon nanopillars, which are capable of vector field steering. By performing mode transformations on both left-handed and right-handed polarization components of the CVBs, cylindrical vector beams can be demultiplexed with high efficiency and reduced crosstalk. Furthermore, by adjusting the phase response of the vectorial diffractive element into a set of Pancharatnam–Berry (PB) phase planes, we experimentally demonstrate 11 CVBs sorting with a set of P–B phase liquid crystal devices. The proposed device may benefit the CVB-based mode multiplexing communications in future.

## 1 Introduction

Cylindrical vector beams with polarization singularities are a type of optical vortices with cylindrical symmetry in polarization [1, 2]. Because of their interesting focusing property, there has been a lot of extensive investigations in singular optics [3, 4], optical trapping [5–8], laser machining [9], microscopic imaging [10, 11], multidimensional optical storage [12], and optical metrology [13]. Apart from that, CVBs are also orthogonal optical modes in both free space and optical fiber, which are capable of providing extra freedom of degree for optical multiplexing communications [14–16]. There are several superiorities with regard to use of CVBs multiplexing in optical communications. Due to their special polarization distributions and eigenmodes property in optical fibers, the CVBs show great advantages in transmission stability and turbulence resilient [17–22]. Through all these years, the generating of CVBs is studied thoroughly, including use of the Mach–Zehnder interference method [23, 24], birefringent laser crystals [25], spatial light modulators [26, 27], metamaterial q-plates [28], and nonlinear processes [29]. Nevertheless, as one key procedure in mode multiplexing communications, the research about the sorting of CVB modes with high efficiency and low crosstalk was insufficient in the past. Recently, some effects have been made in mode (de)multiplexing. Coordinate transformation offers a new approach to mode sorting [30, 31]. A demultiplexing system based on a Cartesian to log-polar transformation was proposed and demonstrated for demultiplexing of orbital angular momentum (OAM) beams [32–36]. Based on this approach, the geometric phase was introduced to distinguish the spin and orbital angular momentum simultaneously [37, 38]. It has also been demonstrated that this method can be used for CVB demultiplexing [39], whereas the overlaps between the sorted CVBs with adjacent topological charges makes the crosstalk an unsolved issue. To reduce the crosstalk between the adjacent modes, a variety of improved methods have been proposed, including separation of the odd and even OAM states into different ports before the Cartesian to log-polar transformation is performed [40]. Some researchers have added a fan-out system to the transformation system to copy multiple plane waves that are adjacent to each other after expansion to obtain more plane wave periods and thus improve the resolution [41–46]. Recently, a new coordinate transformation called the Cartesian-to-spiral transformation was proposed [47], and research shows that the OAM states can obtain more angular periods through this transformation without using a fan-out system, effectively reducing the adjacent OAM mode crosstalk [48–50]. However, these devices are all phase-only elements for OAM sorting and are not suitable for CVB sorting due to the lack of vectorial field response. In order to realize the vectorial field steering, an anisotropic unit cell is required in the device design. In addition, metasurfaces have exhibited polarization-related light field regulation abilities [4, 51], [52], [53], [54], [55], and they have shown promise for use in regulation of vector light fields such as CVBs [56, 57], which can be an ideal solution for the CVBs sorting device.

In this work, we propose a vectorial diffractive optical element that can perform independent vectorial control related to the circular polarization of the light field. Such vectorial optical elements can be realized with metasurfaces or liquid crystal. For the metasurface device, the unit cell is composed of a cylindrical silicon nanopillar on a silica substrate. The phase modulation of two orthogonal circular polarization states in CVBs can be independently controlled with the unit cell. With this design, a lens-less optical coordinate conversion system that capable of independent spiral transformations for the right-handed circular polarization (RCP) and left-handed circular polarization (LCP) components of a CVB is achieved. While adjusting the phase response for the LCP and RCP components as conjugate, the CVBs sorting system can be achieved with a set of liquid crystal Pancharatnam–Berry elements and two Fourier lens. We demonstrate theoretically and experimentally that the system has higher sorting resolution than previous log-pol geometric transformation demultiplexing systems and that it allows the sorting of any optical modes with pair of orthogonal states with linear, circular, or elliptical polarizations.

## 2 Vectorial diffractive optical element design

### 2.1 Vectorial diffractive unit cell design

The vectorial diffraction device possesses the capability to impose independent additional phase modulation to a linearly polarized light field in two orthogonal directions. The rotational transformation of the device’s Jones matrix results in the manifestation of circular polarization-dependent phase modulation, as detailed in the supplementary material. With the characteristics of vectorial diffraction control, such a unit can be used for spatial modulation of the cylindrical vector beam CVB to achieve the function of demultiplexing. This is because the *l*th-order CVB’s polarization state at (*x*, *y*, *z*) can be expressed as follows using the Jones matrix *J*_*l*_:

(1) $$\begin{array}{ll} J_{l} & =E\left(x,y,z\right)\left[\begin{array}{c} \cos\left(l\varphi +\varphi_{0}\right)\\ \sin\left(l\varphi +\varphi_{0}\right) \end{array}\right]\\ & =\frac{E\left(x,y,z\right)}{2}\left[\begin{array}{c} e^{\text{i}\left(l\varphi +\varphi_{0}\right)}+e^{-i\left(l\varphi +\varphi_{0}\right)}\\ \frac{e^{\text{i}\left(l\varphi +\varphi_{0}\right)}-e^{-i\left(l\varphi +\varphi_{0}\right)}}{i} \end{array}\right]\\ & =\frac{E\left(x,y,z\right)}{2}\left(e^{\text{i}\left(l\varphi +\varphi_{0}\right)}\left[\begin{array}{c} 1\\ -i \end{array}\right]+e^{-i\left(l\varphi +\varphi_{0}\right)}\left[\begin{array}{c} 1\\ i \end{array}\right]\right)\\ & ={RCPOAM}_{l}+{LCPOAM}_{-l} \end{array}$$

**Figure 1:**
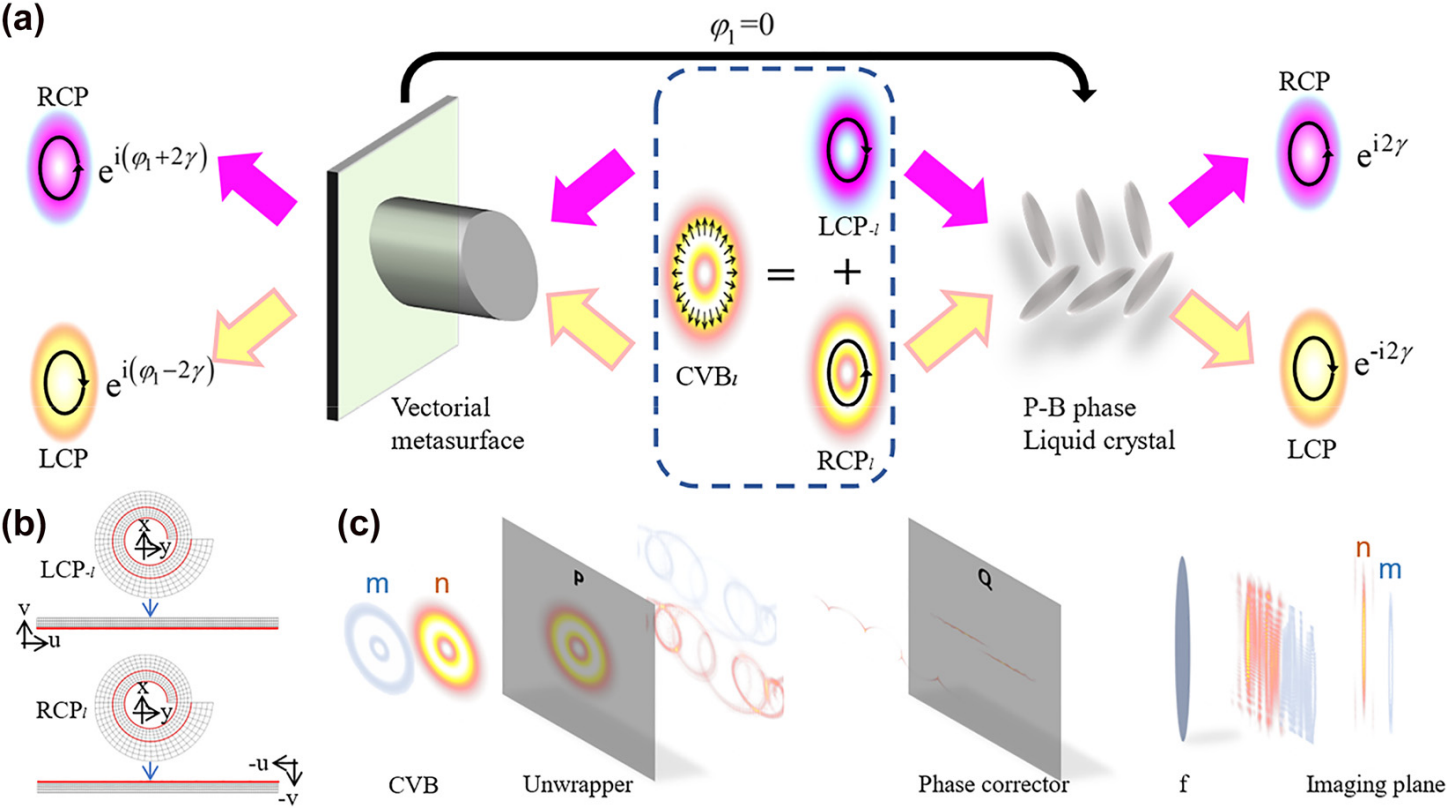
Vectorial diffractive unit cell and optical element design. (a) The *l*th-order of the CVB can be decomposed into a superposition of two OAMs with opposite circular polarizations. With a designed vectorial diffractive unit cell, each polarization state will be modulated with different phase shift with respect to its polarization, and the unit can be vectorial metasurface or P–B phase liquid crystal. (b) Mapping relations and spiral transform process of LCP and RCP components of the CVB. (c) Schematic diagram of the CVB light field conversion process in the case of metasurface. The unwrapper and phase corrector apply a spiral transformation to the incident CVB, and convex lens *f* is used to perform a Fourier transform. When the different orders m and n of the CVB are input, they will be unfolded and sorted as shown by the blue and red patterns, respectively.

In Eq. (1), *E(x, y, z)* represents the amplitude of the CVB at (*x*, *y*, *z*), *φ* is the azimuthal angle at the (*x*, *y*) plane, *φ*_0_ is the initial phase angle, and *l* is an integer. Due to the characteristics of the helical phase terms $e^{\text{i}\left(l\varphi +\varphi_{0}\right)}$ and $e^{-i\left(l\varphi +\varphi_{0}\right)}$ with the circular polarization, Eq. (1) shows that an *l*th-order CVB can be decomposed into two parts, where the RCP component carries the OAM with topological charge l and the LCP component carries the OAM with the opposite topological charge −*l*, as shown in Figure 1(a). When the CVB beam passes through the device composed of the vectorial diffractive unit of Eq. (S2), the light field will be modulated into:

(2) $$\begin{array}{ll} M\cdot J_{l} & =\left[\begin{array}{cc} e^{\text{i}\varphi_{1}}\cos^{2}\gamma +e^{\text{i}\varphi_{2}}\sin^{2}\gamma & \left(e^{\text{i}\varphi_{1}}-e^{\text{i}\varphi_{2}}\right)\cos\gamma \sin\gamma \\ \left(e^{\text{i}\varphi_{1}}-e^{\text{i}\varphi_{2}}\right)\cos\gamma \sin\gamma & e^{\text{i}\varphi_{2}}\cos^{2}\gamma +e^{\text{i}\varphi_{1}}\sin^{2}\gamma \end{array}\right]\\ & \;\times \left(RCP\cdot {OAM}_{l}+LCP\cdot {OAM}_{-l}\right)\\ & =LCP\cdot {OAM}_{l}e^{\text{i}\left(\varphi_{1}-2\gamma \right)}+RCP\cdot {OAM}_{-l}e^{\text{i}\left(\varphi_{1}+2\gamma \right)} \end{array}$$

Eq. (2) shows that the vectorial diffractive device can perform two sets of independent control on the two orthogonal polarization components of the CVB. Therefore, the demultiplexing system must integrate two sets of independent subsystems to control these left-handed and right-handed components separately.

The design of our vectorial diffractive device uses the properties described above and introduces an optical coordinate transformation [27] to deal with the helical phase in the LCP (RCP) light; this approach can expand the azimuth-related tangential phase gradient into a straight-line phase gradient, and demultiplexing of different straight-line phase gradients is then enabled on this basis. Our design uses the spiral coordinate transformation [47], which can obtain more wave numbers than a polar coordinate transformation and can thus reduce crosstalk between adjacent modes after demultiplexing. The spiral coordinate transformation gives the following mapping from plane *P*(*x*, *y*) to plane *Q*(*u*, *v*):

(3) $$\begin{array}{c} u\left(r,\theta \right)=\frac{b}{1+a^{2}}\left[aIn\left(\frac{r}{s}\right)+\theta \right]\\ v\left(r,\theta \right)=\frac{b}{1+a^{2}}\left[\ln\left(\frac{r}{s}\right)-a\theta \right] \end{array}$$

In Eq. (3), *a*, *b*, and *s* are real parameters, $r=\sqrt{x^{2}+y^{2}}$ , and *θ* is the spiral azimuthal angle of (*x*, *y*). Because the two orthogonal circular polarizations in the CVB have opposite vortex phases, to ensure that the phase gradients of these two polarizations are the same after unfolding, the coordinate mappings of the left-hand and right-hand polarizations during the transformation should be RCP: (*x*, *y*) to (*u*, *v*) and LCP: (*x*, *y*) to (−*u*, −*v*); therefore, the phases of plane *P* when applied to the LCP and the RCP should be given, respectively, as:

(4) $$\begin{array}{c} \frac{\partial P_{\text{RCP}}\left(x,y\right)}{\partial x}=k\frac{u-x}{d},\frac{\partial P_{\text{RCP}}\left(x,y\right)}{\partial y}=k\frac{v-y}{d}\\ \frac{\partial P_{\mathrm{LCP}}\left(x,y\right)}{\partial x}=k\frac{-u-x}{d},\frac{\partial P_{\mathrm{LCP}}\left(x,y\right)}{\partial y}=k\frac{-v-y}{d} \end{array}$$

Then, the phase unwrapper for the RCP and LCP can be written as:

(5) $$\begin{array}{ll} P_{\text{RCP}}\left(x,y\right) & =\frac{kb}{d\left(1+a^{2}\right)}\left[\left(ax+y\right)\ln\left(\frac{r}{s}\right)\right.\\ & \;\left.+\left(x-ay\right)\theta -\left(ax+y\right)\right]-\frac{k\left(x^{2}+y^{2}\right)}{2d}\\ P_{\text{LCP}}\left(x,y\right) & =P_{\text{RCP}}\left(-x,-y\right) \end{array}$$

where *θ* in Eq. (5) represents the spiral azimuth angle of (*x*, *y*), and *d* is the distance between the two planes. Through a process of substitution with Eqs. (1) and (2), we can finally find suitable *φ*_1_ and rotation angle *γ* values as follows:

(6) $$\begin{array}{c} \varphi_{1}\left(x,y\right)=\frac{P_{\text{RCP}}\left(x,y\right)+P_{\text{RCP}}\left(-x,-y\right)}{2}\\ \gamma \left(x,y\right)=\frac{P_{\text{RCP}}\left(x,y\right)-P_{\text{RCP}}\left(-x,-y\right)}{4} \end{array}$$

Similarly, in the phase correction plane, the phase distributions of the RCP and LCP are:

(7) $$\begin{array}{ll} Q_{\text{RCP}}\left(u,v\right) & =\frac{kbs}{d\left(1+a^{2}\right)}e^{\frac{au+v}{b}}\cdot \left[\sin\left(\frac{u-av}{b}\right)\right.\\ & \;\left.+a\cos\left(\frac{u-av}{b}\right)\right]-\frac{k\left(u^{2}+v^{2}\right)}{2d}\\ Q_{\text{LCP}}\left(u,v\right) & =Q_{\text{RCP}}\left(-u,-v\right) \end{array}$$

The corresponding *φ*_1_ and rotation angle *γ* are given by:

(8) $$\begin{array}{c} \varphi_{1}\left(x,y\right)=\frac{Q_{\text{RCP}}\left(u,v\right)+Q_{\text{RCP}}\left(-u,-v\right)}{2}\\ \gamma \left(x,y\right)=\frac{Q_{\text{RCP}}\left(u,v\right)-Q_{\text{RCP}}\left(-u,-v\right)}{4} \end{array}$$

**Figure 2:**
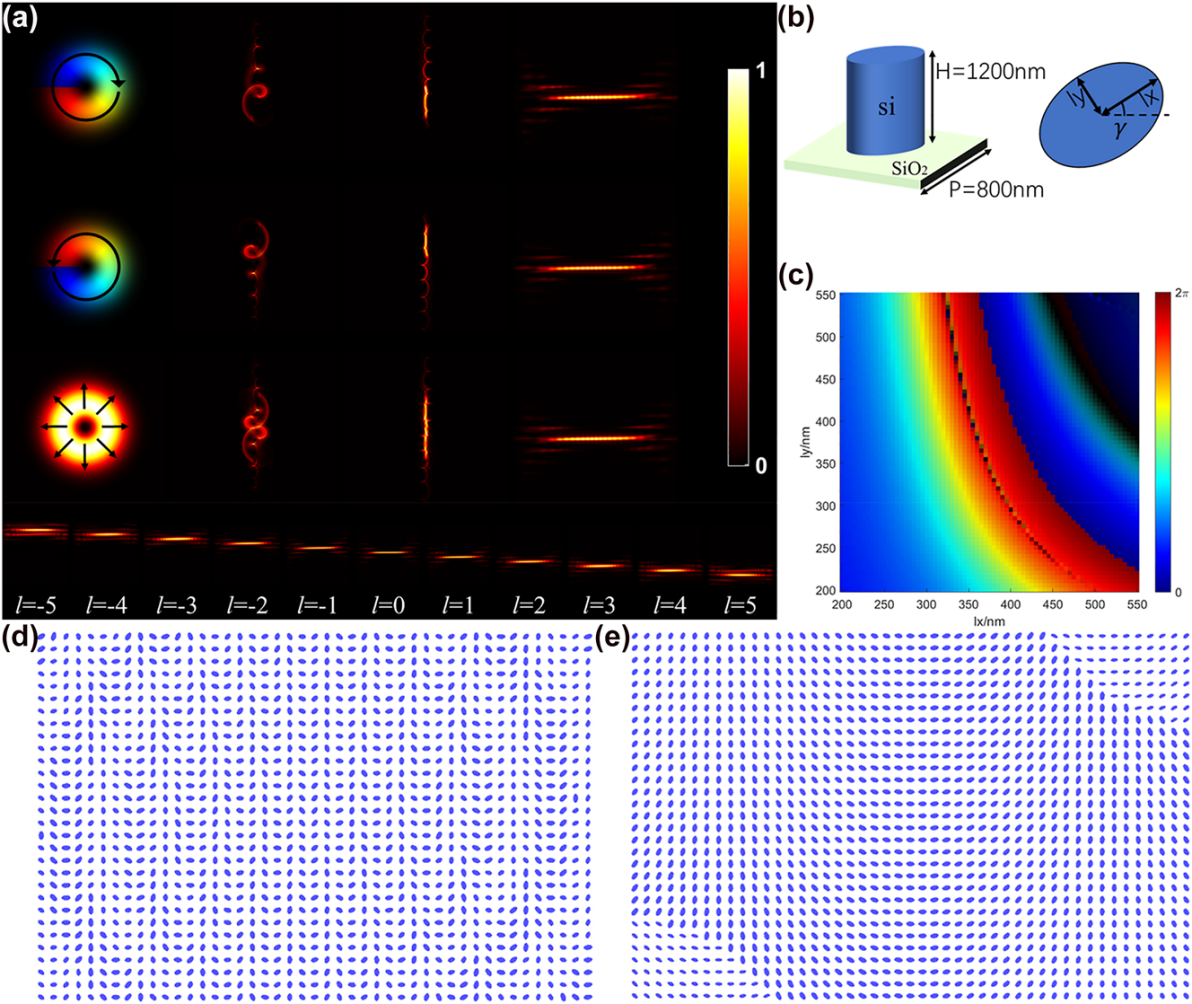
CVB demultiplexing process and the designed metasurface devices. (a) Transformation process of the LCP and RCP components and the total field intensity, along with the demultiplexing results for the different orders of the CVB; black arrows indicate the polarization direction. (b) A unit cell of the metasurface is composed of a silicon elliptical cylinder on a silicon dioxide substrate. The height of the cylinder is 1200 nm, and the pitch of the unit cell is 800 nm. (c) Calculated phase modulation of a unit cell with respect to its long axis *lx* and short axis *ly*, while the long axis is along the *x* direction. (d) and (e) Central areas of the designed metasurfaces *P* and *Q*, respectively.

At this stage, the two spin circular polarization components of the CVB can undergo opposite spiral expansion processes between *P* and *Q* and are then emitted after phase correction. After the same-order CVB is expanded, the LCP and RCP components have the same phase gradient, which means that after focusing through the lens, the two components can converge to the same position. At the same time, the different orders of the CVB have different phase gradients; this means that they converge to different horizontal positions, and thus CVB demultiplexing is realized. Note that during this process, the phases applied by *P* and *Q* to the left-hand and right-hand polarization components at the same point are independent, i.e., two sets of coordinate transformation systems are integrated. Figure 2 shows the demultiplexing process for the CVB beam. In Figure 2(a), the transformation processes for both the left-handed component and the right-handed component are shown, along with the transformation process for the total light field. We can clearly see that in the first two rows, the left-handed and right-handed circular polarization components of CVB are first gradually expanded into straight lines along the opposite helical rotation directions, completing independent coordinate transformations. After that, it is focused by an optical lens to form a linear spot at the same position on the focal plane of the lens. The sorting process for the single-order CVB total field strength is given in the third row. The demultiplexing results for the orders −5 to 5 of the CVB are also shown at the bottom of Figure 2(a).

Such vectorial diffractive device can be constructed by controlling the shape and rotation angle of the metasurface unit cell. The metasurface is composed of a silicon dioxide substrate and a fixed-height elliptical silicon column, as illustrated in Figure 2(b). The calculation results presented in Figure 2(c) show that selection of the appropriate major axis *lx* and minor axis *ly* can satisfy the phase modulation *φ*_1_ condition calculated using Eqs. (6) and (8), and then rotation by an angle *γ* is performed to obtain the location of the unit structure; the final metasurface is composed of a series of elliptical cylinder arrays with differing rotation angles and axes of unequal lengths.

When a lens is added between *P* and *Q*, the phase gradient condition for the transformation system becomes:

(9) $$\begin{array}{c} \frac{\partial P_{\text{RCP}}\left(x,y\right)}{\partial x}=\frac{u}{d},\frac{\partial P_{\text{RCP}}\left(x,y\right)}{\partial y}=\frac{v}{d}\\ \frac{\partial P_{\text{LCP}}\left(x,y\right)}{\partial x}=\frac{-u}{d},\frac{\partial P_{\text{LCP}}\left(x,y\right)}{\partial y}=\frac{-v}{d} \end{array}$$

This shows that the unwrapper phase gradients of the LCP and RCP components are opposites, which conforms to the case in which *φ*_1_ = 0 in Eqs. (S3) and (S4). The Jones matrix then simplifies to:

(10) $$\left[\begin{array}{cc} \cos2\gamma \left(x,y\right) & \sin2\gamma \left(x,y\right)\\ \sin2\gamma \left(x,y\right) & -\cos2\gamma \left(x,y\right) \end{array}\right]$$

At this time, the phase adjustment can be realized using the PBOE, and *γ* becomes the rotation direction of the fast axis. Finally, the Pancharatnam–Berry (PB) phase modulations of the device on plane *P* and plane *Q* are given, respectively, as:

(11) $$\begin{array}{ll} 2\gamma_{P} & =-\frac{kb}{\left(1+a^{2}\right)f_{1}}\left[\left(ax+y\right)In\left(\frac{r}{s}\right)+\left(x-ay\right)\theta -\left(ax+y\right)\right]+\frac{kL}{f_{1}}y \end{array}$$

(12) $$2\gamma_{Q}=\left\{\begin{array}{c} \frac{kbs}{\left(1+a^{2}\right)f_{1}}e^{\frac{au+v}{-b}+\frac{L}{b}}\left[\sin\left(\frac{u-av}{-b}-\frac{aL}{b}\right)+a\cos\left(\frac{u-av}{-b}-\frac{aL}{b}\right)\right]+\frac{kL}{f_{1}}v-\frac{kL}{f_{2}}v,v>0\;\\ \frac{kbs}{\left(1+a^{2}\right)f_{1}}e^{\frac{au+v}{b}+\frac{L}{b}}\left[\sin\left(\frac{u-av}{b}-\frac{aL}{b}\right)+a\cos\left(\frac{u-av}{b}-\frac{aL}{b}\right)\right]+\frac{kL}{f_{1}}v-\frac{kL}{f_{2}}v,v<0\; \end{array}\right.$$

In Eq. (11), the first term plays the role of spiral coordinate transformation; light incident on plane *P* will expand along the spiral into a horizontal straight line on the *Q*-plane, and while the LCP and RCP components expand in opposite directions, the second term separates the LCP and RCP components in the vertical direction to become two parallel lines located *L* apart from the center. Plane *Q* plays the role of compensating for the phase modulation of plane *P* and causes the LCP and RCP components to recombine in the same place after lens 2 focuses them on the focal plane. The demultiplexing system is shown in Figure 4(a); all elements are coaxial, the phase unwrapper (plane *P*) is located at the focal plane of lens 1, the phase corrector (plane *Q*) is located at the common focal plane of lens 1 and lens 2, the imaging plane is located at another focal plane of lens 2, and the input CVBs are incident vertically along the axis.

### 2.2 PBOE devices

**Figure 3:**
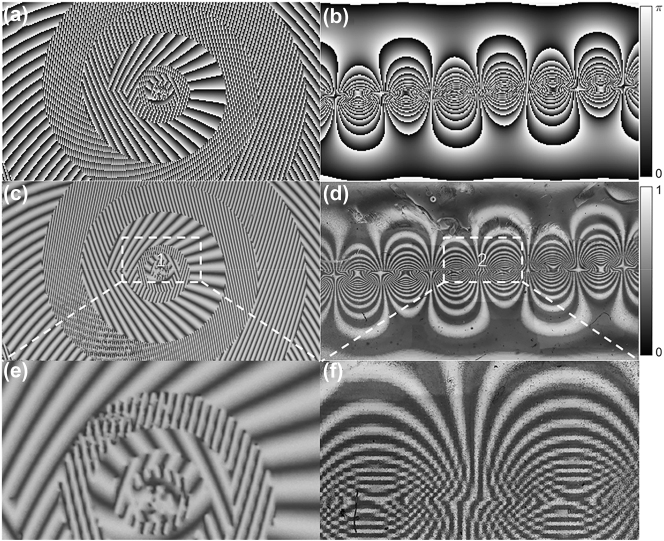
PB-phase and liquid crystal molecule fast axis orientation of the PBOE device. (a) and (b) Geometric phase (twice the rotation angle of the fast axis of the liquid crystal molecules) distributions of the unwrapper, i.e., the phase corrector device. (c) and (d) Patterns of the processed device in (a) and (b), respectively, when observed under a microscope. (e) and (f) Partial areas 1 and 2 in parts (c) and (d), respectively.

The PBOE device at the *P* and *Q* birefringent surfaces is composed of birefringent liquid crystal molecules that are fixed on the surface of the glass substrate. We adjust the thickness of the liquid crystal layer to ensure that the two polarization components are incident along the fast axis and the slow axis after transmission to produce an *λ*/2 optical path difference. At the same time, the angle between the fast axis of the liquid crystal molecules on the plane and the horizontal direction is also adjusted point-by-point by using a digital micromirror device maskless lithography system, with values equal to *γ*_*P*_ and *γ*_*Q*_ for the two polarization components. The device size is 5 mm by 10 mm, containing 180 by 320 pixels with a pixel size of 30 μm. Figure 3 shows the designed PBOE devices, where Figure 3(a) (Figure 3(b)) shows the PB-phase of P(Q), and also shows the fast axis orientation distribution of the liquid crystal molecules. Figure 3(c) and (d) show diagrams produced by observation of the processed device under a microscope. Figure 3(e) and (f) show areas 1 and 2 in Figure 3(c) and (d), respectively.

## 3 Results and discussion

**Figure 4:**
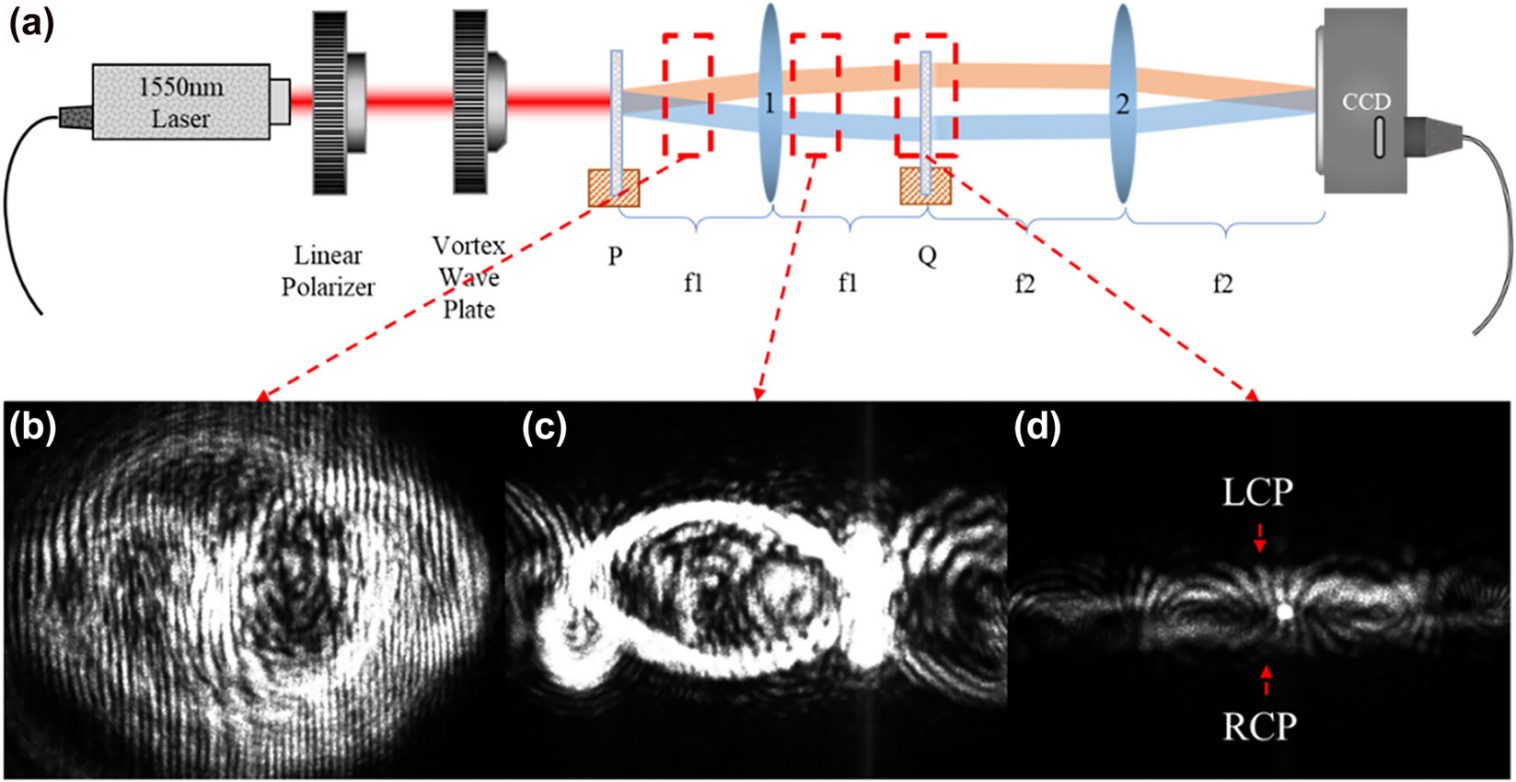
Schematic diagram of the optical setup used for the CVB demultiplexing system. (a) A 1550 nm laser generates the CVB after passing through a linear polarizer and a vortex wave plate, devices *P* and *Q*, and lens 1. Total light intensities passing through (b) the unwrapper and (c) lens f1. (d) Light intensities of the LCP and RCP components after the spiral transform.

In this section, we built a sorting system using two optical lenses and the two liquid crystal devices prepared in the previous section. We set wavelength *λ* as 1550 nm, the focal length *f*_*1*_ of lens 1 is 15 cm, the focal length *f*_*2*_ of lens 2 is 20 cm, *L* = 0.7 mm, *a* = ln[1.5/(2π)], *b* = 2.9/(2π) mm, and *s* = 1.3 mm. The input order of the CVBs is from −5 to 5. Figure 4 shows a schematic diagram of the optical setup for the proposed system. A collimated beam is emitted from a 1550 nm laser and converted into linearly polarized light after passing through a linear polarizer; a vortex wave plate causes this beam to become a CVB for use as the input signal, and an infrared charge-coupled device (CCD) receives the sorting light. Figure 4(d) shows the LCP and RCP components of the CVB, indicating that the incident left and right rotations have performed the spiral transformation as expected. We input single orders of the CVB one at a time, in order from −5 to 5, and the CCD records the output light location at the imaging plane.

**Figure 5:**
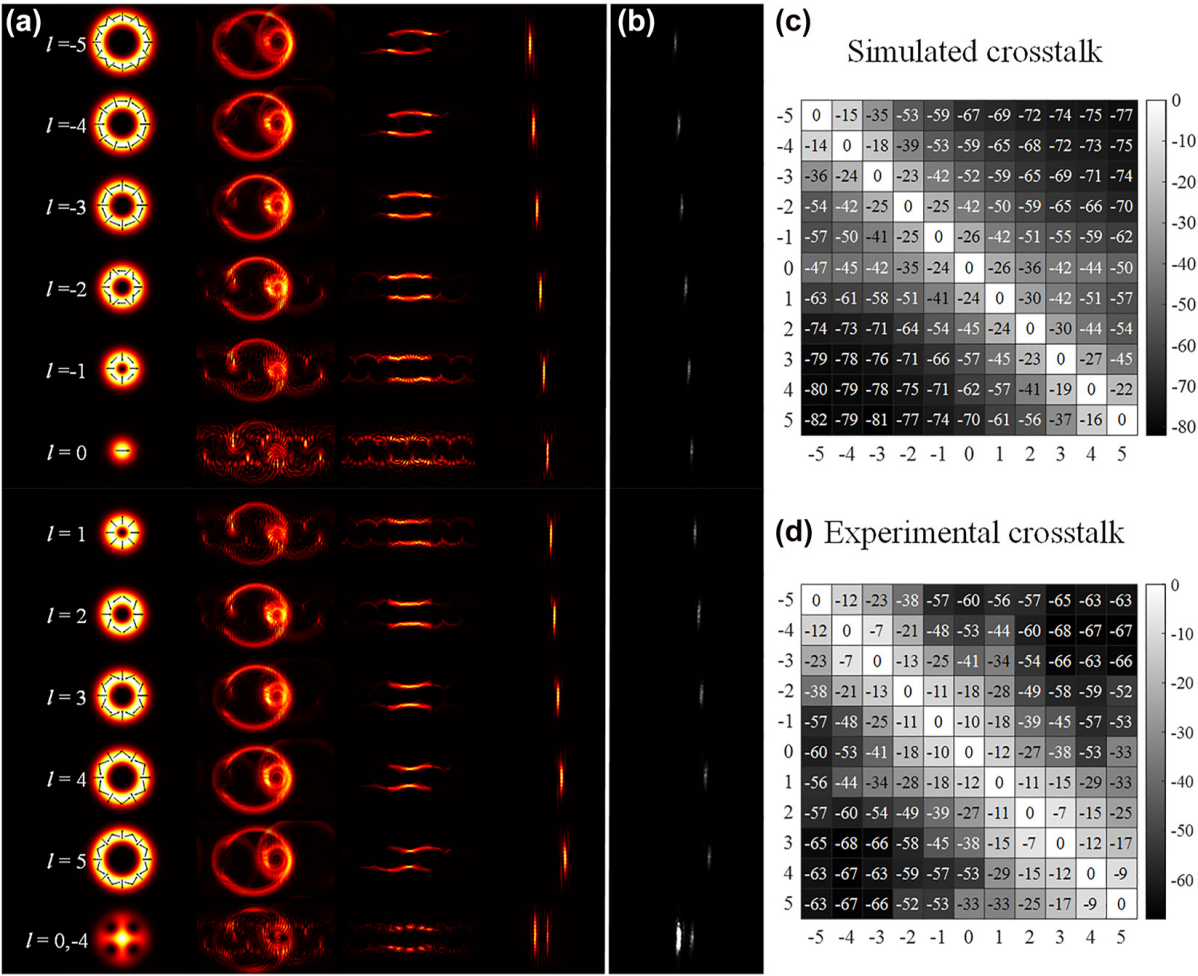
Theoretical and experimental results. (a) Theoretical calculations of orders −5 to 5 of the CVB intensity during the transformation and demultiplexing process. (b) Experimental demultiplexing results for orders −5 to 5 of the CVB modes. The sorting results for orders 0 and −4 of the CVB are also shown in the bottom row. (c) The numerical simulation and (d) experimentally demonstrated crosstalks are also given, respectively.

Figure 5 shows a comparison of the experimental results with the theoretical results, where Figure 5(a) is the calculated sorting results for the different orders of the CVB, and the corresponding experimental results are shown in Figure 5(b). The incident CVBs are arranged horizontally in the sequence of their CVB order, which fits well with the numerical simulations. Figure 5(c) and (d) are the corresponding crosstalk of simulation and experiment, respectively. They are obtained by calculating the ratio of the energy of other orders CVB to the focal point at the position of the preset CVB mode. The results show that the simulation and experimental results of the maximum crosstalk between adjacent order crosstalk are about −14 db and −7db, and better crosstalk can be obtained when the disparity of the orders is getting larger.

**Figure 6:**
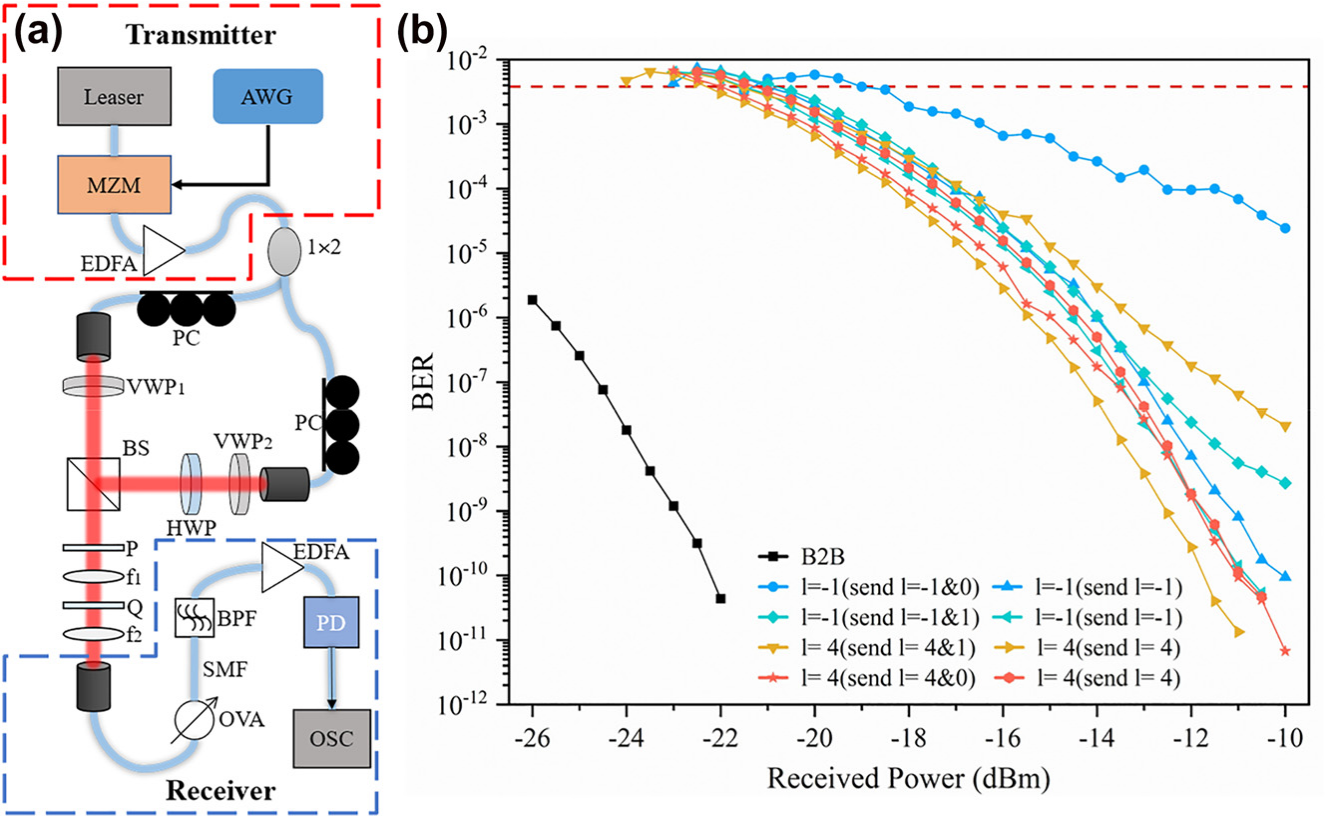
Two-way coaxial CVB demultiplexing optical communication. (a) Schematic diagram of the experimental setup, including the signal transmitter, the demultiplexing system, and the receiver. (b) The measured bit error rate of the target channel after demultiplexing of the two-channel CVB group signal; the horizontal dotted line represents the error correction threshold.

We also demonstrate the free space demultiplexing of the system under two coaxial CVB optical communication signal inputs. Figure 6(a) shows a communication experimental setup where the signal is demultiplexed at the receiving end and then connected to the optical fiber. The 1550 nm laser beam is modulated to carry a 10Gbit/s on–off key signal. After amplification, it is divided into two lines by a coupler and a polarization controller for polarized light collimation and output to free space and then converted into CVB mode by vortex wave plates of different orders. Then the two signals are combined into a coaxial beam by a beam splitter. After passing through our sorting system, the target signal is coupled to a single-mode fiber for bit error measurement. Figure 6(b) shows the measured bit error rate (BER) curve, and the curve labeled B2B represents the back-to-back transmitted signals of the system without mode multiplexing. In order to test the performance difference between adjacent orders in mode multiplexing communications, there are four groups of multiplexed CVB signals of *l* = 0 & −1, *l* = 1 & −1, *l* = 1 & 4, and *l* = 0 & 4. The measured EBR of the target signal marked with different colors. At the same time, as a reference, the measured EBR of single-channel signal transmission is also illustrated in Figure 6(b), and they are marked with the same color as the corresponding multiplexed signal group. The experimental results show that with the multiplexed two CVB beams in the same group of signals, the difference of the order decreases, the deviation of the EBR and the B2B increases, which indicates that the crosstalk is larger when the order of the CVBs is similar. Due to the limitations of current fabrication accuracy of the devices, we were only able to verify CVB mode sorting from −5 to 5th order in our experiments. However, by enlarging the size of the devices and optimizing the fabrication accuracy, it is possible to perform CVB mode sorting with the order up to 20, which means that the multiplexing modes number can be up to 40.

## 4 Conclusions

In summary, we have demonstrated a vectorial diffractive optical system to sort the orders of CVBs. Through the first two planes in the system, the LCP and RCP components of the CVB are realized via independent inverse spiral transformations, and the metasurface structure is designed accordingly. Subsequently, by adding lenses between the first two transformation planes, the transformation is simplified to the PB phase alone, and under this condition, liquid crystals are used to process the device. We introduce spiral coordinate transformations and polarization-dependent modulation, causing the LCP and RCP components of the CVB to expand and separate in opposite directions, and later merge together after phase correction. We have verified the function of the system with lower crosstalk through both simulations and experiments from orders −5 to 5 of the CVB. Finally, we modulate multiple sets of two coaxial CVB signals for communication testing to verify the availability of the demultiplexing system. We believe that this research will have important applications in the optical mode multiplexing communication.

## Supplementary Material

Supplementary Material Details
