# Supplementary material for: Cylindrical vector beams demultiplexing communication based on a vectorial diffractive optical element
Source: Nanophotonics. 2023 Mar 17;12(9):1753–62. doi: 10.1515/nanoph-2023-0009 (PMC11501804; doi:10.1515/nanoph-2023-0009)
Supplement: Supplementary file 1 — Supplementary Material Details [file j_nanoph-2023-0009_suppl_001.docx]

Supplementary Materials for “Cylindrical vector beams demultiplexing communication based on a vectorial diffractive optical element”

Derivation of circular polarization independent phase modulation:

A vectorial diffractive device should have different modulation responses to light fields with different polarization directions. First, the Jones matrix for a vectorial diffractive device that can add independent phase control in two orthogonal linear polarization directions can be written as:

(S1)

In Eq. (S1),is the phase applied to the linearly polarized light along the x-axis, andis the phase applied to the polarized light along the y-axis. When it is rotated by an angle of$\gamma$relative to the x direction, the Jones matrix is transformed into:

(S2)

In special cases such as that where , when the LCP light passes through the device, the following applies:

(S3)

For the RCP light, the following applies:

(S4)

From Eq. (S3) and Eq. (S4), by adjusting the rotation angle$\gamma$and the phase, the device can produce independent phase modulation of the incident light and convert LCP (RCP) light into RCP (LCP) light.


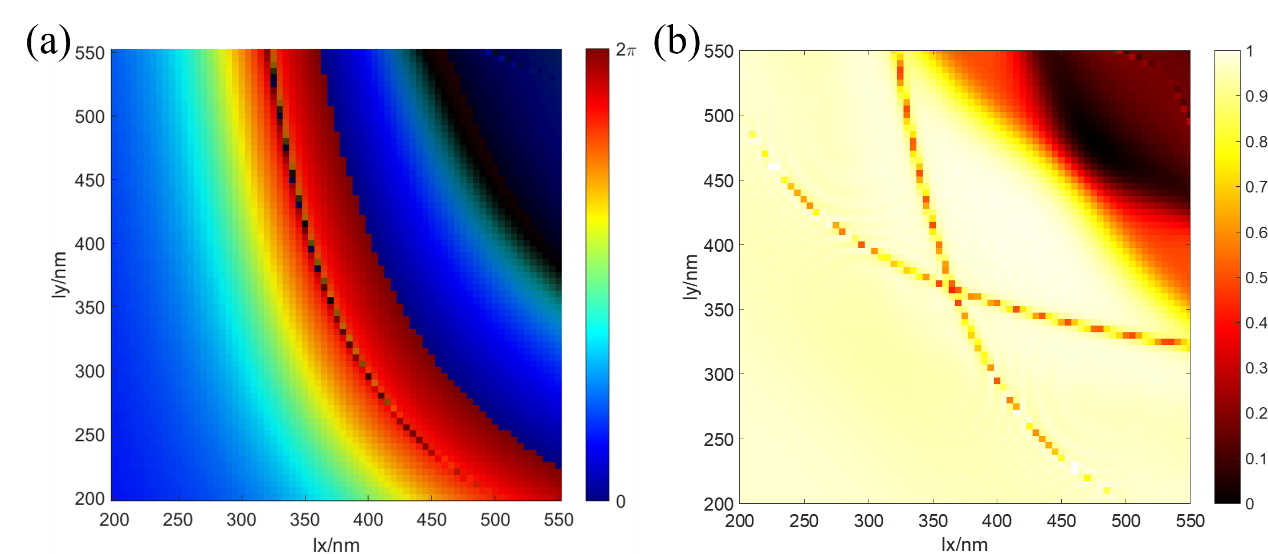


Fig. S1: The modulation phase (a) and the transmittance (b) of an unit cell of the metasurface with respect to the length of the short/long axis.


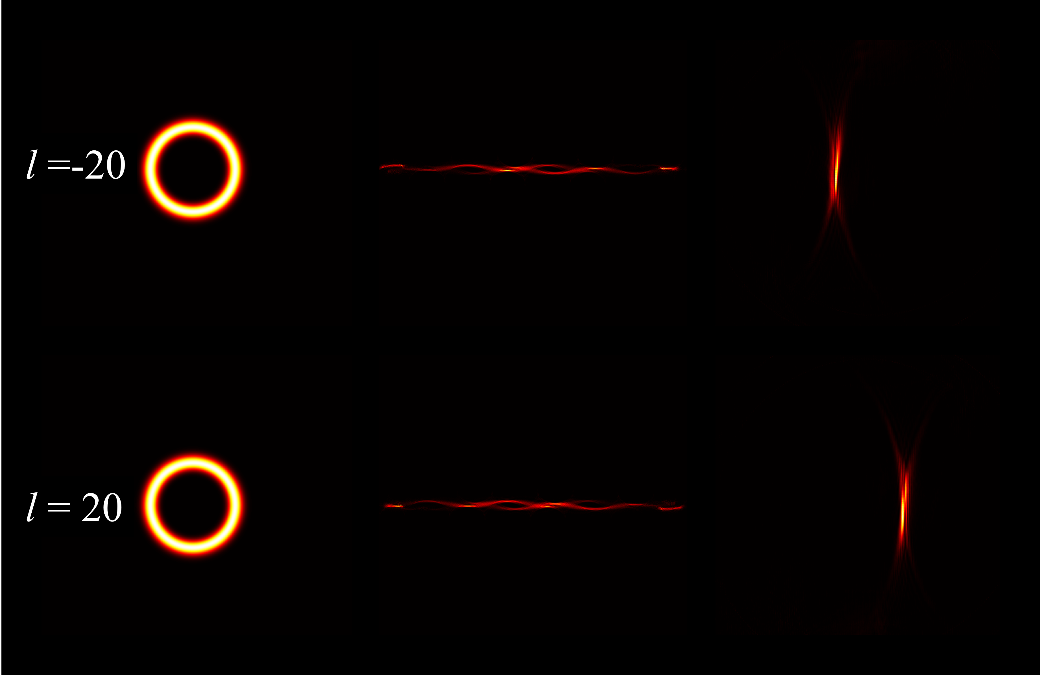


Fig. S2: The numerical demonstration of the sorting of -20 order and 20 order CVB with larger device size and higher precision of PB phase modulation.
